# Case of class II lupus nephritis with nephrotic features in the context of belimumab: Intersecting clinical challenge with academic initiative to emphasize social barriers in care

**DOI:** 10.1177/2050313X251336061

**Published:** 2025-05-13

**Authors:** Ahyeon Cho, Joshua Shoemaker, Ahmad Matarneh, Sundus Sardar, Pankhuri Mohan, Erik Washburn, Amanda Karasinski, Gurwant Kaur, Nasrollah Ghahramani

**Affiliations:** 1 Pennsylvania State University College of Medicine, Hershey, PA, USA; 2 Division of Nephrology, Department of Medicine, Penn State Milton S. Hershey Medical Center, PA, USA; 3 Department of Medicine, Penn State Health Milton S. Hershey Medical Center, PA, USA; 4 Department of Pediatrics, Penn State Health Milton S. Hershey Medical Center, PA, USA; 5 Division of Pathology, Penn State Health Milton S. Hershey Medical Center, PA, USA

**Keywords:** lupus nephritis, class II, belimumab, nephrotic syndrome

## Abstract

Lupus nephritis is a severe complication of systemic lupus erythematosus that can cause significant kidney damage, leading to symptoms such as proteinuria, hematuria, and renal failure. If not managed promptly, it can progress to chronic kidney disease or even end-stage renal disease. Early diagnosis and treatment are vital to prevent such outcomes. While traditional treatments focus on immunosuppressive therapies, the introduction of belimumab offers a new treatment approach. Kidney biopsies play a crucial role in diagnosing and classifying lupus nephritis, helping guide appropriate treatment. Class II lupus nephritis typically requires supportive care, but more severe cases demand aggressive therapy. Noncompliance with treatment can worsen the condition, highlighting the need to address social challenges that impact patient care and adherence. We hereby report a 19-year-old female with systemic lupus erythematosus and nephrotic-range proteinuria, diagnosed with Class II lupus nephritis shortly after initiating belimumab, highlighting the social challenges impacting her care and follow-up. We hereby report a 19-year-old female known with systemic lupus erythematosus with nephrotic-range proteinuria and Class II lupus nephritis. Diagnosed shortly after belimumab, and highlighting the social challenges in her care and follow-up.

## Introduction

Lupus nephritis (LN) affects ~40% of individuals with systemic lupus erythematosus (SLE).^1^ This condition involves the deposition of immune complexes in the glomerulus, leading to inflammation and potential damage. These immune deposits disrupt the normal function of the kidneys, resulting in symptoms such as proteinuria and hematuria. If not managed properly, the condition can lead to more severe kidney issues, including chronic kidney disease or end-stage renal disease.^2^

The revised International Society of Nephrology/Renal Pathology Society classifies LN into six distinct classes based on kidney biopsy results.^3,4^ Class II LN, also known as mesangial proliferative LN, is characterized by immune deposits primarily in the mesangial areas of the kidneys, with minimal involvement elsewhere. This classification helps guide treatment decisions, with Class II LN typically requiring less aggressive treatment compared to other classes.^5^

In this case, a 19-year-old woman with a history of SLE was diagnosed with Class II LN shortly after starting belimumab for her underlying lupus. Her degree of proteinuria was severe and more than the degree that would be expected in Class II LN. This change suggests that belimumab might have altered the progression of her disease, resulting in kidney biopsy findings consistent with Class II LN rather than a more advanced classification, leading to a more severe presentation. In addition, this case highlights significant social challenges that impacted her ability to manage her condition effectively. Social challenges have significantly impacted the patient’s care. This highlights the need for comprehensive care approaches addressing both medical and social needs as it would result in improved outcomes.

## Case presentation

A 19-year-old female with a history of SLE presented to the emergency department with severe swelling throughout her body. Diagnosed with SLE at age 15, she had a complex medical history marked by positive autoimmune markers and fluctuating complement levels. Over the years, she had been treated with various immunosuppressive drugs, most recently transitioning to azathioprine 75 mg once daily. By early 2024, she struggled with medication adherence, leading to increased levels of dsDNA antibodies and decreased complement levels. Her difficulties included transportation issues, communication barriers, and missed appointments.

**Figure 1.**
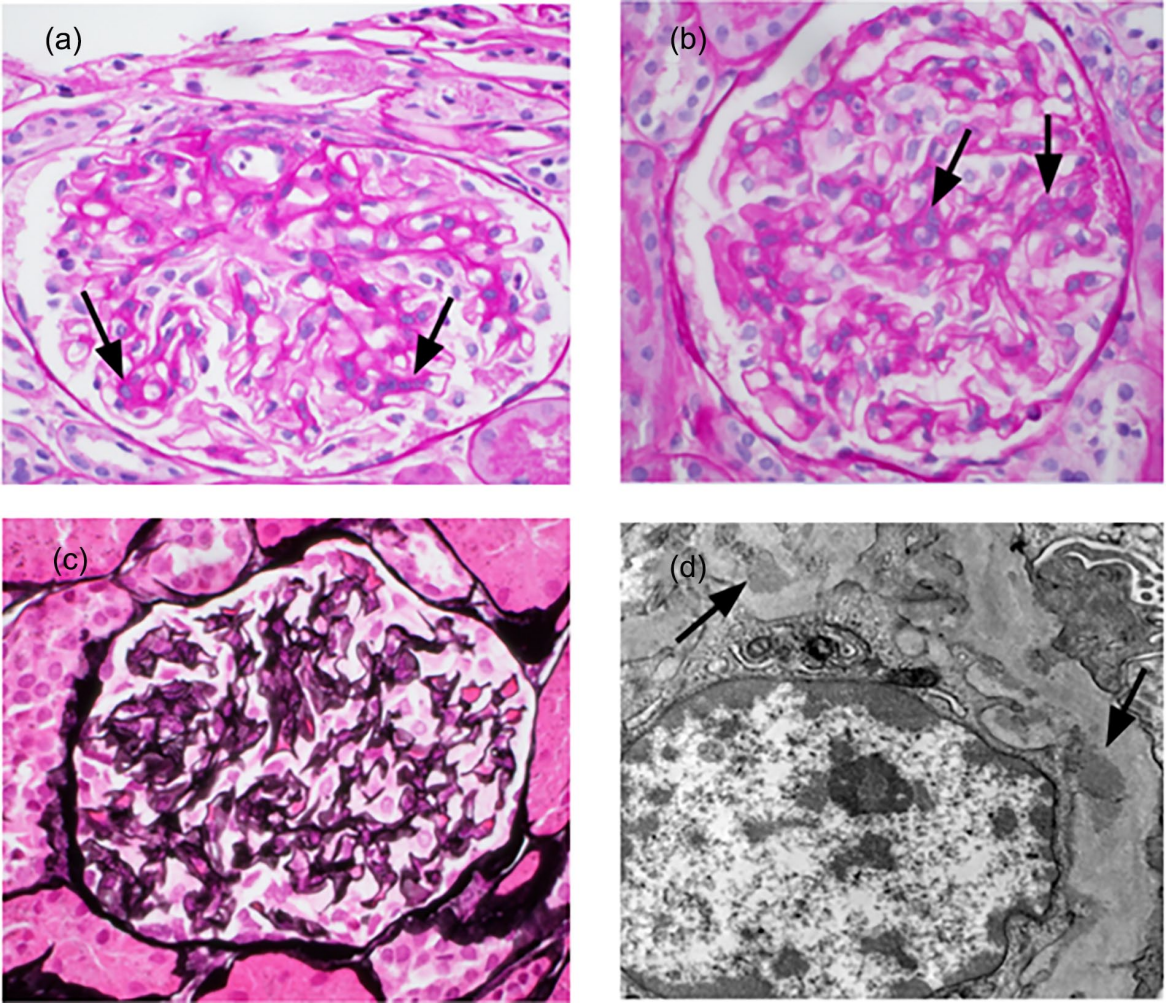
(a, b) The glomeruli show mesangial hypercellularity and matrix expansion (arrows) with no endocapillary hypercellularity, hyaline deposits, neutrophils/karyorrhexis, or cellular/fibrocellular crescents (PAS, 600×). (c) The silver stain shows mild basement membrane Glomerular basement membrance (GBM) thickening with no epimembranous spikes or GBM duplications. (Jones silver stain; 600×). (d) Electron microscopy images reveal mesangial deposits (shown; arrows) as well as subepithelial and subendothelial electron-dense deposits (not shown).

**Figure 2.**
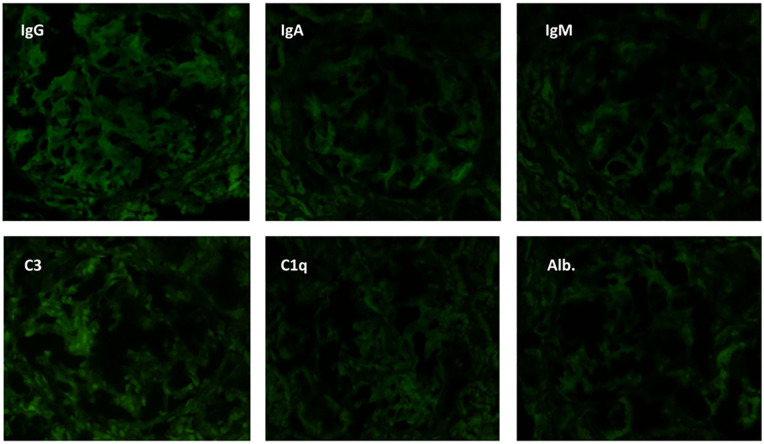
Immunofluorescence reveals a “full house” pattern with glomerular staining for IgG (4+), IgA (3+), IgM (3+), C3 (4+), and C1q (1+) mainly in the capillary wall and less in the mesangial area. There is negative staining for albumin.

In March 2024, she began taking belimumab 600 mg IV while continuing on a daily dose of prednisone 5 mg. By April 2024, she was admitted to the hospital with nephrotic syndrome and leukopenia. Despite a kidney biopsy confirming Class II LN, the high level of proteinuria suggested that the disease might be more severe. The biopsy showed segmental sclerosis, mesangial expansion, and mild thickening of the basement membrane, with electron microscopy revealing extensive podocyte damage consistent with Class II LN (Figures 1 and 2). Table 1 shows laboratory findings on presentation.

**Table 1.** Laboratory findings on presentation.

Laboratory value	Reference range	On presentation
Hemoglobin	13–17 g/dl	9.3
White blood cells	4–10 k/µl	310.67
Platelets	150–300 k/µl	469
Creatinine	0.7–1.3 mg/dl	0.68
BUN	6–23 mg/dl	7
ALT	0–33 µ/l	9
AST	0–32 U/l	13
Albumin	3.5–5.2 g/dl	2
Urine PCR	<150 mg/g	20.3 g/g

ALT: alanine transaminase; AST: aspartate aminotransferase; BUN: Blood Urea Nitrogen.

Even with treatment, including steroids and mycophenolate mofetil, she continued to miss follow-up appointments and struggled with medication adherence. By June 2024, her condition worsened, and she returned to the emergency department with severe edema, fatigue, and vomiting. She had stopped all medications a few days before this visit due to feeling unwell and had received three doses of belimumab. Her physical examination revealed significant swelling and lab tests showed very low albumin levels and severe proteinuria. Imaging identified fluid accumulation in her chest and abdomen. She was treated with high-dose intravenous methylprednisolone, furosemide, and albumin. Upon discharge, her treatment plan was adjusted to include a higher dose of prednisone and a plan to increase mycophenolate mofetil, with close monitoring.

The patient was followed up in the outpatient setting within a week of discharge. She reported significant lower extremity edema. However, she was consuming a diet high in sodium, which contributed to urinary retention despite adherence to furosemide and prednisone as recommended. The patient did not increase her mycophenolate mofetil dose as advised, which further contributed to the complexity of her care.

Repeat laboratory tests showed normal renal function with a creatinine of 0.7 mg/dl and an estimated glomerular filtration rate of over 90 ml/min/1.73 m^2^, with no electrolyte abnormalities. However, she continued to experience an active flare, marked by hypoalbuminemia and proteinuria. We collaborated closely with her pediatric rheumatologist and maintained regular contact through phone calls and portal messages, monitoring her labs every 2 weeks.

## Discussion

Class II LN typically presents with mesangial expansion and mild proteinuria, not the severe nephrotic syndrome observed in this patient. Nephrotic syndrome, characterized by significant proteinuria, is more commonly associated with Class V LN, which involves more diffuse damage to the glomerular capillary walls and subepithelial deposits.^6^ The unusual presentation in this case, with extensive podocyte damage and severe proteinuria despite the biopsy showing Class II LN, suggests a complex interaction that may involve the new treatment regimen.^7^

Belimumab, a monoclonal antibody targeting B-cell activating factor, represents a newer approach to treating LN by reducing the survival of autoreactive B-cells and decreasing autoantibody production. Its introduction has provided an additional tool for managing LN, especially for patients with refractory disease.^8^ However, the unexpected findings, in this case, raise questions about how belimumab might influence the progression of LN or alter its classification.^9^

The possibility that belimumab might downplay the biopsy in nephrotic syndrome is intriguing. Belimumab’s effects on the immune system could impact immune complex deposition and podocyte function, potentially leading to atypical presentations.^10^ The extensive podocyte damage observed in this patient is rare for Class II LN and might suggest that belimumab’s impact on immune regulation could have contributed to findings a less severe biopsy finding that was encountered in this patient or could have contributed to the more severe presentation of Class II LN.^11^

Another consideration is the potential for belimumab to interact with other treatments or underlying disease mechanisms in ways not fully understood. In this case, despite adding belimumab to her treatment regimen, the patient’s ongoing medication noncompliance and social challenges may have further complicated her disease manage-ment. This highlights the importance of considering both pharmacologic effects and real-world factors when assessing patient outcomes.^12^

Social determinants of health, such as socioeconomic status, access to healthcare, and social support, play a crucial role in the management of LN and SLE.^13^ This patient faced significant barriers, including difficulties with transportation, communication issues, and missed appointments, which impacted her ability to adhere to treatment and follow-up care. These social challenges not only hindered her treatment adherence but also likely contributed to her disease progression and worsening symptoms.

Addressing these social determinants requires a multifaceted approach. Providing practical support such as transportation assistance and financial aid, coupled with enhanced patient education and engagement, is essential for improving adherence and health outcomes.^14^ Tailoring support systems to individual patient needs can help bridge gaps in care and address barriers to effective management.^15^

Moreover, this case underscores the importance of a collaborative approach in managing complex conditions such as LN. Effective communication and coordination among healthcare providers, including primary care physicians, rheumatologists, nephrologists, and social workers, are crucial. An interdisciplinary team can address both the medical and social aspects of patient care, ensuring a comprehensive and supportive treatment plan. By integrating clinical management with support for social challenges and ongoing research, healthcare providers can enhance the management of LN and improve the quality of life for patients facing this complex condition.

## Conclusion

This case illustrates the complexities and challenges of managing LN, especially with the advent of new treatments such as belimumab. The unexpected biopsy findings and severe nephrotic syndrome highlight the need for detailed monitoring and personalized treatment plans. In addition, addressing social determinants of health is crucial for improving disease management and patient adherence. By combining clinical care with support for social challenges, healthcare providers can significantly enhance patient management and well-being. A comprehensive approach that integrates both medical and social support is essential for achieving the best outcomes in patients with LN.
